# Crystallinity, Rheology, and Mechanical Properties of Low-/High-Molecular-Weight PLA Blended Systems

**DOI:** 10.3390/molecules29010169

**Published:** 2023-12-27

**Authors:** Hongwei Yang, Jianghua Du

**Affiliations:** 1 School of Materials Science & Engineering, North Minzu University, Yinchuan 750021, China; 2 Key Laboratory of Polymer Materials & Manufacturing Technology, North Minzu University, Yinchuan 750021, China

**Keywords:** polylactic acid, melt blending, crystallization behavior, rheological behavior, diluent, nucleating agent

## Abstract

As semi-crystalline polyester (lactic acid) (PLA) is combined with other reinforcing materials, challenges such as phase separation, environmental pollution, and manufacturing difficulties could hinder the benefits of PLA, including complete biodegradability and strong mechanical properties. In the present investigation, melt blending is utilized to establish a mixture of low- and high-molecular-weight polylactic acids (LPLA and HPLA). The crystallinity, rheology, and mechanical properties of the combination were analyzed using rotational rheometry, differential scanning calorimetry, X-ray diffraction, polarized optical microscopy, scanning electron microscopy, and universal testing equipment. The results demonstrate compatibility between LPLA and HPLA. Moreover, an increase in LPLA concentration leads to a decrease in the crystallization rate, spherulite size, fractional crystallinity, and XRD peak intensity during isothermal crystallization. LPLA acts as a diluent during isothermal crystallization, whereas HPLA functions as a nucleating agent in the non-isothermal crystallization process, promoting the growth of LPLA crystals and leading to co-crystallization. The blended system with a 5% LPLA mass fraction exhibits the highest tensile strength and enhances rheological characteristics. By effectively leveraging the relationship between various molecular weights of PLA’s mechanical, rheological, and crystallization behavior, this scrutiny improves the physical and mechanical characteristics of the material, opening up new opportunities.

## 1. Introduction

One of the most crucial factors that define the microstructure of polymers and affect their physical properties is the behavior of the disordered phase during crystallization to produce the ordered structure during the material molding process. Researchers have continually focused on polymer crystallization research, which has attracted attention in an extensive range of disciplines, from basic science to polymer processing and application. Crystallization dynamics affect polymer crystallization, crystal size, and crystal shape. The change in crystal structure is also capable of affecting the nucleation mechanism, surface free energy, and chain arrangement [1,2,3,4,5]. Understanding polymer crystallization, from both theoretical and practical points of view, is paramount for designing product macrostructures and regulating final properties.

Bio-based plastics will reportedly increase from 2.2 million tonnes in 2022 to 6.3 million tonnes in 2027. As is commonly understood, PLA is a type of biodegradable polyester material that is semi-crystalline in nature and has been dubbed “green plastic”. Decent mechanical and thermal stability, coupled with eco-friendly features such as biodegradability, composability, and recyclability, renders PLA one of the most favorable substitutes for petrochemical-based plastics. It is worth mentioning, however, that the shortcomings of PLA, such as its high brittleness (unsuitable for high-stress plastic deformation applications) and its very slow crystallization and degradation rate, have to some extent prevented its widespread commercialization and development [6,7,8,9]. For this reason, various approaches have been attempted to enhance its mechanical, crystalline, and processing properties; in particular, chain expansion and blending techniques are often adopted to improve melt strength [10,11,12]. Blending PLA with other polymers is regarded as an efficient approach to broaden its application areas, including poly(ethylene oxide) (PEO) [13,14,15], poly(hydroxybutyrate) (PHB) [16,17,18], and poly(caprolactone) (PCL) [19,20]. Additionally, other PLA-based blends have been documented. Wang et al. [21] utilized tetrabutyl titanate to obtain a block copolymer of poly(lactic acid) and poly(propylene carbonate) through ester exchange. The process resulted in enhanced system compatibility and overall performance of the copolymer. Fu et al. [22] explored the biodegradation dynamics of PLA/PBAT mixtures in freshwater and sediments and established that the blend’s crystallinity amplified with time and was inversely related to the degradation.

However, it appears that the aforementioned techniques are not particularly effective for achieving phase separation and interfacial bonding in materials within multiphase systems. The causes of this phenomenon include the limited conformational plasticity of PLA, molecular chain migration challenges, and insufficient time for molecular chain reorganization, which leads to the formation of amorphous states during non-isothermal crystallization [6]. Therefore, investigators have sought to raise the crystalline nucleation point by introducing conventional inorganic nucleating agents like talc [23] and graphite [24]. These agents serve to help nucleation or accelerate the growth of spherical crystals [7,25]. Wang et al. [25] co-extruded PLA filled with nucleating agent TMC200 to establish a multilayered structure. As a result, this induced a high content of continuous and ordered transverse crystals. These crystals responded positively to the improvement of mechanical strength. It is worth mentioning that incorporating nucleating agents into PLA can compromise its biodegradability and alter its molecular weight, resulting in a substantial reduction in mechanical strength and other detrimental outcomes.

Material properties are noticeably influenced by crystal structure and morphology. Therefore, it is crucial to regulate the final properties of polymer materials by optimizing crystallization conditions and investigating the correlation between structure and properties. According to the literature, tiny crystalline regions dispersed within an amorphous matrix are able to effectively inhibit the thermal movement of molecular chains and physically entangle molecular chain segments around the crystalline regions. By employing the crystals as physical network points, they could contribute to the strength of PLA melt [26]. This study systematically explores the thermal, rheological, and mechanical properties of two distinct molecular-weight blends of polylactic acid (PLA) based on various techniques, including differential scanning calorimetry (DSC), X-ray diffraction (XRD), polarized optical microscopy (POM), scanning electron microscopy (SEM), rotational rheometry, and a universal mechanical tester. This work aims to methodically examine the crystallization process and crystal morphology of the systems, with various mass ratios, and enhance the potential of PLA development in new areas of application. These new areas include fully degradable materials, such as food packaging, foams, construction, and drug carriers.

## 2. Results and Discussion

### 2.1. Non-Isothermal Crystallization and Thermal Properties of Blended Systems

**Figure 1:**
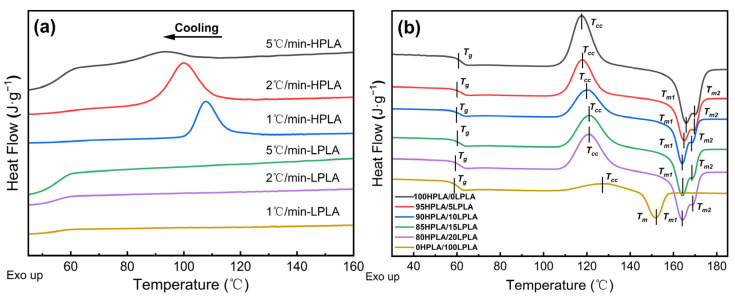
DSC curves for pure PLA and HPLA/LPLA blending systems: (**a**) non-isothermal plots for various cooling rates, and (**b**) second heating curves.

**Figure 2:**
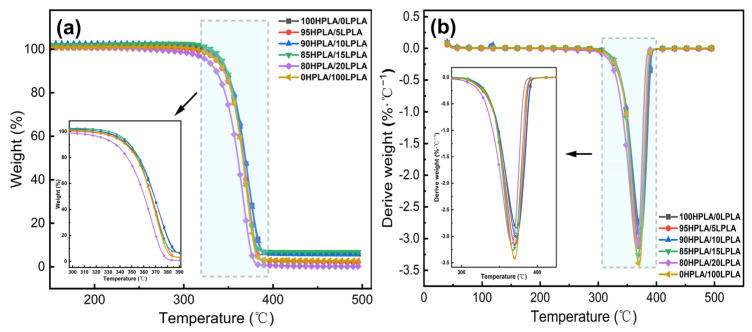
(**a**) TGA plots, and (**b**) DTG plots of both the pure PLA and HPLA/LPLA binary blended system in the presence of the N_2_ atmosphere.

In light of this, the mechanical, rheological, and thermal properties of the chain structure, crystal structure, and blend phase structure created by the crystalline polymer blend system are intimately related to the characteristics of the polymer, and this exhibits a significant impact on its crystallization behavior [27,28]. The DSC is a useful exploration technique for analyzing the thermal dynamics of two different molecular order PLA and mix systems [29]. Figure 1 illustrates the DSC curves of pure PLA and HPLA/LPLA blends. Figure 2a displays the system’s DSC curves at various cooling rates, Figure 1b demonstrates the system’s DSC curves at 10 °C/min following cooling, and Table 1 presents the related thermodynamic characteristics.

As indicated in Figure 1b, the DSC curve of the blend system has a glass transition temperature, denoted by *T_g_*, which indicates that the two polymer blend systems are compatible [30]. More evidence can be provided for the Cole–Cole and Han curves implemented in the rheological study. The component ratio of the system affects the cold crystallization temperature (*T_cc_*) and melting temperature (*T_m_*), which are between those of the LPLA and HPLA. Furthermore, since the only difference between the LPLA and the HPLA is their molecular weights and their chemical formula and content are the same, both the LPLA-based and HPLA-based polymers can be reasonably considered compatible, meaning that the interfacial tension is approximately equal to zero [31]. In addition, the DSC curves presented in Figure 1a of pure HPLA and cooling pure LPLA at different cooling rates show that the HPLA exhibits distinct crystallization peaks at various cooling rates. The crystallization temperature (*T_cp_*) gradually rose as the cooling rate decreased to a high temperature, and the peak shape became sharper. However, the LPLA is a smooth and straight line and does not exhibit exothermic phenomena due to crystallization, indicating that crystallization of the LPLA occurs at a significantly slower rate than the HPLA under non-isothermal conditions. When combined with the cold crystallization peak in pure LPLA in Figure 1b, a wide temperature range appears, indicating that the free energy of the crystalline system is low, the nucleation process is difficult, the crystallization is incomplete, and the molecular chain untangling and dynamic equilibrium of heavy entanglement is not clear.

Do the crystallization behaviors of components in compatible LPLA and HPLA compounds influence each other? As presented in Table 1, with the increase in LPLA content in the blend system, the cold crystallization temperature (*T_cc_*) gradually changed to a high temperature, the initial melting temperature (*T_onset_*) gradually changed to a low temperature, and Δ*H_cc_* and Δ*H_m_* of the blend system can be considered constant within the experimental error range. Under the same conditions, *H_cc_* and *H_m_* for the pure LPLA in order are 30.90% and 22.57% of the blended system. If the LPLA is considered to be unsolidified because its crystallization rate is much slower than that of the HPLA, Δ*H_cc_*, and Δ*H_m_* of the blended system should lessen with increasing the LPLA content. However, the actual results show that Δ*H_cc_* and Δ*H_m_* of the blending system are not correlated with the mass ratio of the LPLA in the system, which reveals that the crystallization behavior of the LPLA also occurs in the system, and the LPLA and HPLA are completely compatible, so co-crystallization occurs in the blending system.

The *T_cc_* of the blend system increased with the content of the LPLA and was placed between the two pure PLAs (117.61–128.18 °C), and its half-peak width *T*_(1/2)*w*_ progressively increased, indicating that the addition of the LPLA incorporated into the reduction of the crystallization rate of the HPLA, the regularity of chain segments, and the crystallization capacity. In the blending system, the LPLA acts as a diluent. The higher melting point of the HPLA leads to the growth of the crystallization rate of the LPLA, mainly acts as a nucleating agent, and accelerates the crystallization growth of the LPLA. The double melting peak of the blend system increased dramatically with increasing the LPLA content, and the melting temperature was similarly reduced with the growth of the LPLA content. The melting recrystallization of the polymer was essentially caused by the premature melting of partial crystals formed at a relatively low temperature and the difference in the degree of crystallinity during the cooling process [32,33]. This shows that the LPLA components have a particular effect on the crystallization perfection of the blend system. The solidus of the double melting peak (the extrapolated initial melting temperature *T_onset_* corresponding to the first peak) ranged from 160.7 °C for pure HPLA to 146.4 °C for pure LPLA and the liquidus (second peak temperature) from 170.3 °C for pure HPLA to 151.8 °C for pure LPLA. No significant discrepancy in the enthalpy value of the blend was observed, indicating that the total heat absorbed and released in the crystallization and melting process is approximately the same, and there is no significant decrease in enthalpy, which exhibits a positive significance in practical application. Additionally, the results revealed that the enthalpy values of the blends also did not substantially alter. According to the positive correlation between the degree of crystallinity and the enthalpy of melting, this may be the reason why the crystallinity of the blend system remains almost constant.

Thermogravimetry is commonly regarded as a useful tool for investigating the relationship between material structure and thermal stability, as well as the thermal change process of materials. The thermal breakdown process for most biomass materials may be divided into four stages: drying and water loss, transition, rapid pyrolysis, and carbonization [34]. The TGA and first-order microquotient DTG graphs of both the pure PLA and binary mixed systems in the N_2_ environment are illustrated in Figure 2. The DTG feedback liquid or solid–gas phase transition causes weight loss in terms of temperature [33].

As can be seen from Figure 2 and Table 2, the pure PLA and the blended systems have a thermal decomposition reaction step with an identical shape within the experimental temperature range, and the melting peak temperature does not alter much. When the temperature rises to 350 °C, the TG curve exhibits a more obvious accelerated weight loss phenomenon, and all systems demonstrate relatively good thermal stability and display high heat resistance.

As a summary, compatible blends of LPLA and HPLA are capable of reducing the crystallization rate of high components under low-molecular-weight non-isothermal crystallization conditions while increasing the capacity of low-molecular-weight crystallization. In the blended system, low-molecular-weight PLA and high-molecular-weight PLA are co-crystallized. Meanwhile, all systems have good heat resistance.

### 2.2. Crystal Structure of the Blending System

**Figure 3:**
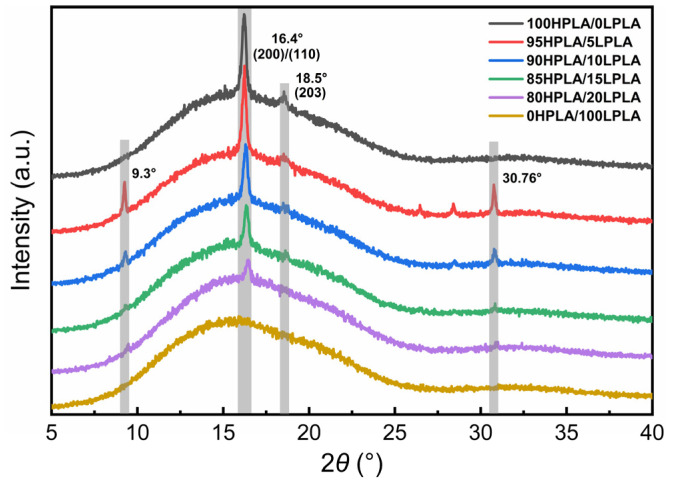
XRD patterns of the pure PLA and HPLA/LPLA blended systems.

Polymer crystallization is a discontinuous phase transition, and the molecular chain exhibits long-range diffusion, which is effectively the folding of the chain segment. The essential variables that define the crystal structure are the chain structure and chain behavior, and the molecular weight division and distribution directly affect the crystallization of PLA, so the crystallization behavior is strongly related to the crystal morphology [35]. The most common crystal forms of PLA are α crystal and α’ crystal, a quasi-orthogonal crystal system, with different formation mechanisms and promotion strategies [36]. The XRD pattern of the samples produced from the HPLA, LPLA, and HPLA/LPLA blend system after complete melting at a specific pressure and temperature of 180 °C and holding at 80 °C for 5 min is illustrated in Figure 3.

The plotted XRD pattern reveals that the LPLA is a diffuse amorphous steamed bread peak, while the HPLA and the blended system have obvious sharp peaks at 2*θ* = 16.4°, indicating that no crystallization occurs in the LPLA subjected to the XRD sample preparation conditions, while pure HPLA and the mixed system can be crystallized. The distinct peak at 2*θ* = 16.4° corresponds to the typical diffraction peak of PLA crystal, whose crystal structure is α or α′ crystal, and the characteristic peak at 18.5° (203) crystal plane exhibits a weak peak [37,38]. The analysis was performed on the crystallinity and peak intensity of X_c-XRD_ at 2*θ* = 16.4°. The peak intensity and crystallinity of the blend system both decreased with increasing the LPLA content, indicating that the LPLA has a diluting effect on the HPLA. As a result, the HPLA molecules were involved in the network at a slower rate, and other molecular fragments were occupied before they had a chance to generate nearby folded fragments [39,40]. In addition, two new diffraction peaks of the HPLA and LPLA appear at 2*θ* = 9.3° and 30.76°, revealing that both the LPLA and HPLA crystallize in isothermal crystallization at 80 °C [41,42], but the effect of co-crystallization on the crystallinity of the system is weaker than the dilution effect of the LPLA on the HPLA.

### 2.3. Crystal Morphology of the Blending System

**Figure 4:**
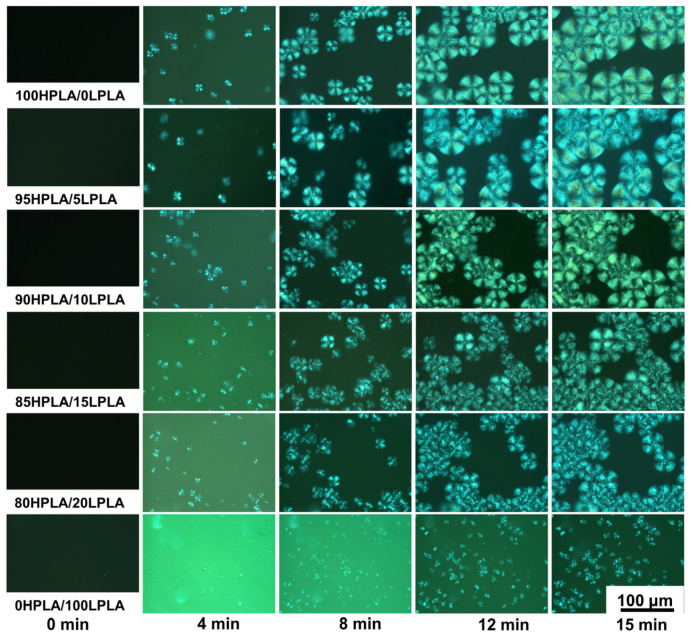
Crystal morphologies of the pure PLA and HPLA/LPLA blended system crystallized isothermally at 120 °C for 15 min.

The polymer crystallization process is generally divided into two stages: nucleation and growth [43]. The POM investigated the isothermal crystallization of the mixtures at various group ratios at 120 °C since the *T_cc_* of practically all samples in Table 1 is at 120 °C. The crystal forms of the HPLA, LPLA, and HPLA/LPLA during isothermal crystallization at 120 °C are presented in Figure 4. For each sample group, five observation time points (0, 4, 8, 12, and 15 min) were selected. The experimentally observed results demonstrate that during the POM observation time, the HPLA crystals and the HPLA/LPLA hybrid system have a black cross-shaped spherulite morphology, with the HPLA spherulite size being the largest and decreasing with the growth of the LPLA content. Only the fine-grained morphology of the LPLA can be seen in the figure, with no obvious black “cross” spherulites, signifying that the crystallization rate of the LPLA is substantially lower than that of HPLA under isothermal crystallization conditions at 120 °C, and the addition of LPLA reduces both the crystallization rate and growth size of HPLA spherulites. According to the above data and Figure 4, the LPLA exhibits a lower nucleation ability than the HPLA. The addition of the LPLA to the HPLA leads to a diluting effect and the amorphous component inhibits the formation of crystalline components and affects the spherulite growth rate and crystallization ability of the HPLA, which is consistent with DSC and XRD data [28,43].

### 2.4. Rheology of the Blending System

**Figure 5:**
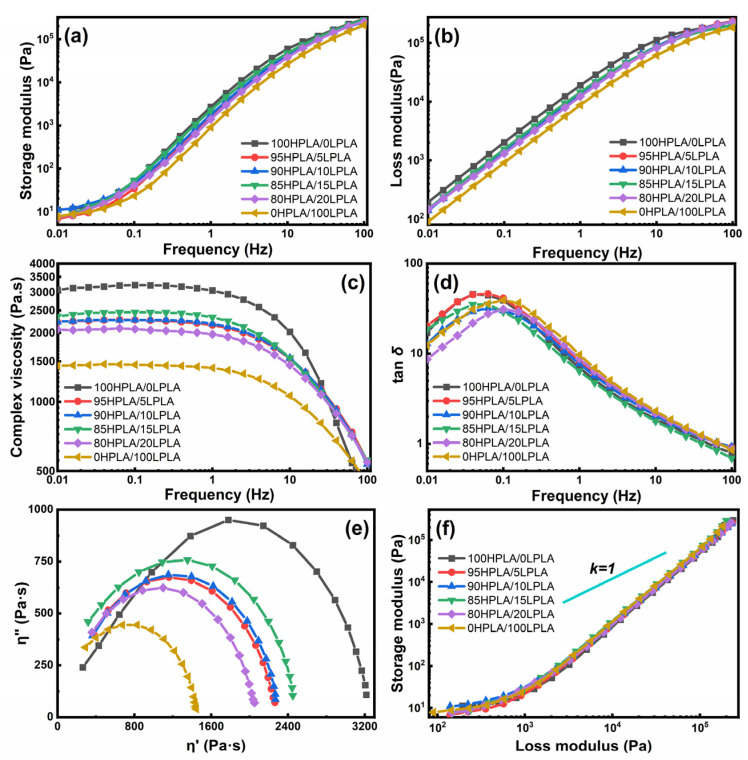
Rheological characteristics curves of both the pure PLA and HPLA/LPLA blended systems: (**a**) storage modulus, (**b**) loss modulus, (**c**) complex viscosity, (**d**) loss tangents, (**e**) Cole-Cole plots and (**f**) Han plots.

The rheological parameters of the polymer melt govern the flow state and shape of the final product, as well as the crystallization methodology [31]. Since the rheological behavior of polymers is determined by the molecular surface structure and interfacial contact, the study of rheological properties is very pivotal for the processing properties of polymers, the strength of joint interaction, and the structure and properties of composite materials [31,44]. The obtained curves of energy storage modulus (*G*′), loss modulus (*G*″), complex viscosity (η*), loss factor (*tanδ*), and frequency (*ω*) based on the dynamic rheological test mode at 180 °C are illustrated in Figure 5.

The *G*′ and *G*″ of the blend system are between the *G*′ and *G*″ of the LPLA and HPLA in the whole frequency range, and the composition of the blend system has no apparent effect on its value and the trend of the curve change trend. This indicates that different mass ratio components do not have a substantial effect on the viscoelasticity of the blend system in the small deformation range, as illustrated in Figure 5a,b. The complex viscosity of the blended system reduces with the growth of the LPLA composition, as shown by the η*-*ω* relationship in Figure 5c, and it is also in the range of the complex viscosity of the LPLA and HPLA. In addition, the melt viscosity of the blend system increased with *ω*, and the viscosity reduction trend of the complex at high frequency was similar to that of the LPLA, indicating that LPLA has a significant effect on the viscosity and shear thinning of the HPLA. The factor *Tanδ* is also a vital characteristic to describe the relaxation behavior of viscoelastic materials [45]. The *tanδ* − *ω* relation in Figure 5d shows that practically all systems have *tanδ* values greater than 1, indicating that *G*″ > *G*′ and the system is substantially dominated by the viscous motion. According to published data, the polymer melt exhibits a negative slope in this curve, but the solid state demonstrates a positive slope [46]. The graph shows the change in slope from positive to negative, indicating the transition from solid to melt. The lower the *tanδ*, the faster the melt’s elastic response and the larger its elasticity. The *tanδ* of the blended system lessens with increasing the LPLA concentration, which is consistent with the rheological law of viscosity reduction, indicating that the system is homogeneous. For instance, the LPLA and HPLA are compatible systems within the experimental composition range, which is consistent with the DSC results. The greatest curvature radius of the pure HPLA corresponds to the highest viscosity, whereas the smallest curvature radius of the pure LPLA corresponds to the lowest viscosity. In addition, there were no tails or two semicircles due to irregular relaxation time [47]. The Han curve, presented in Figure 5f, is usually exploited to evaluate the compatibility of the polymer mixtures [48]. The Han curves of the mixed systems in the figure are superimposed on each other, showing a homogeneous arch curve and gradually approaching the diagonal line, indicating that the system has a homogeneous structure and an enhanced elastic response. The high-frequency region is essentially linear and lies to the lower right of the diagonal slope of 1, displaying that *G*″ dominates the overall mechanical behavior of the system. After linear fitting, it is discovered that the slopes of the mixed systems are all 1.74, which is almost close to the ideal value of 2, and the same slope also indicates that the blending property of the systems is good [48,49].

### 2.5. Morphology and Structure of the Blending System

**Figure 6:**
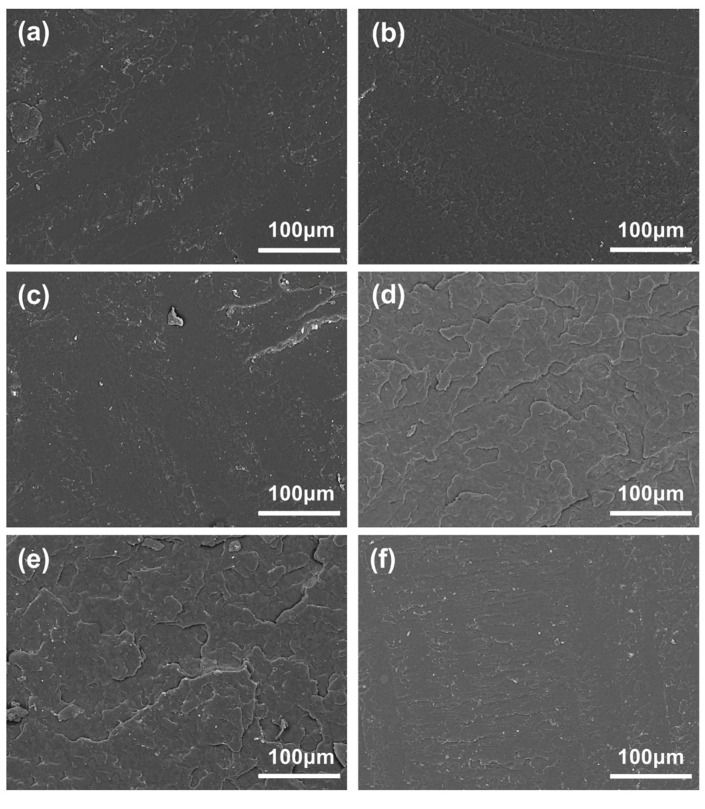
SEM micrographs of various samples: (**a**) 100HPLA/0LPLA, (**b**) 95HPLA/5LPLA, (**c**) 90HPLA/10LPLA, (**d**) 85HPLA/15LPLA, (**e**) 80HPLA/20LPLA, (**f**) 0HPLA/100LPLA.

Figure 6 depicts the phase morphology of the impact fracture surface of the pure PLA and blended system samples. The SEM photographs show that the fracture surface of the samples with the LPLA content less than 10% (mass fraction, as below) in the blended system has a flat and smooth single-phase morphological characteristic. However, when 15% LPLA is added, the fracture surface of the blend becomes rough. When the content of LPLA increased to 20%, the cross-section even showed a stepped morphology with a stratification trend. This may be chiefly related to the dilution effect of the LPLA, which reduces the viscosity of the system (measured by analyzing the rheological properties) and the phase separation process caused by crystallization [28]. The interfacial morphology of the HPLA/LPLA blend is very similar to other low-toughness polymer/rubber blends [50], and the SEM images confirm the results of the previous investigations.

### 2.6. Mechanical Properties of the Blending System

**Figure 7:**
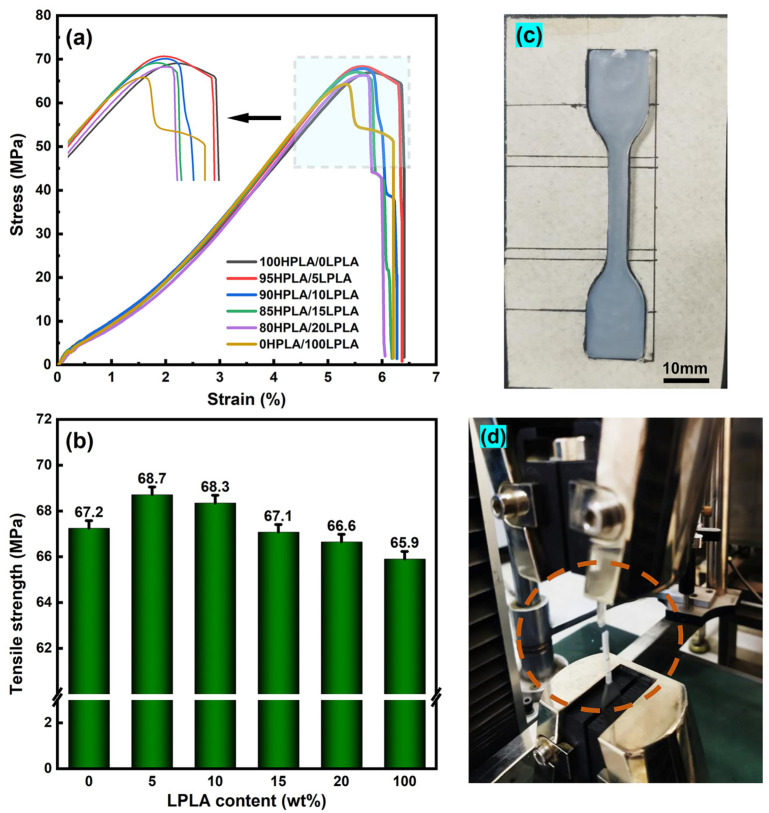
Mechanical properties of PLA blending system: (**a**) stress–strain curves, (**b**) tensile strength as a function of the LPLA content, (**c**) stretch of the spline, (**d**) tensile fracture.

Tensile mechanical tests were performed on samples from various systems to methodically examine the direct effect of the two molecular weight fractions on the mechanical properties of the materials. The stress–strain curves of the HPLA, LPLA, and HPLA/LPLA blends are presented in Figure 7. As shown in Figure 7a, the HPLA, LPLA, and HPLA/LPLA blend systems all exhibit typical brittle fracture characteristics during the tensile test, demonstrating hardness and strength, with fracture stresses of less than 6.5%. It is worth noting that when the composition of the blend system is greater than 5%, it exhibits certain ductile fracture characteristics. However, the breaking strength is lower than that of LPLA and the plastic deformation is reduced, which can be attributed to the higher crystallinity of the composite system than of the LPLA (see Section 2.1 for DSC and Section 2.2 for XRD analysis). Compared to the HPLA, the tensile strength of the blends first demonstrated an ascending trend and then a descending trend with growth of the LPLA percentage, with the 5LPLA blends having the highest tensile strength of all blends at 68.7 MPa. Even when the LPLA mass ratio rises to 15%, the tensile strength of the blend would be almost the same as that of the pure HPLA, which indicates that the LPLA does not affect the mechanical properties of the blended system. Figure 7c,d illustrate the instantaneous state of tensile spline and tensile fracture. The tensile strength due to the influence of low molecular weight decreased gradually, and the tensile modulus lessened after a maximum value in the range, indicating that the LPLA played a relative role in toughening, and the toughness of the system enhanced to some extent. It is well understood that polymer crystallization plays a pivotal role in mechanical properties, and polymer chain folding essentially relies on polymer dynamics as well as properties. As analyzed in the previous section, the nucleation and growth of polymer crystals could be also affected by the addition of low-molecular-weight and good-flowability LPLA, which is ultimately manifested in the improvement of mechanical properties [51].

### 2.7. Mechanism Analysis

**Figure 8:**
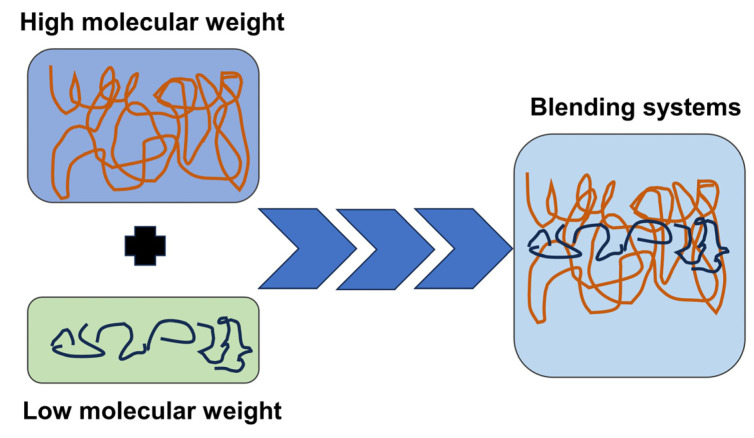
Schematic representation of the mechanism analysis.

Polydisperse molecular weight polymers show preferential crystallization of long chains in the crystalline region, as evidenced by the phenomenon of molecular segregation. High-molecular-weight HPLA exhibits higher crystallinity, more visible crystallinity peak in the DSC curve, and higher relative viscosity, whereas LPLA possesses a low molecular weight, high melt flow rate, and low viscosity. The motion of molecular chain segments during melting and blending enhances the number of physical entanglement points, and the small crystalline region dispersed in the amorphous matrix could effectively limit the thermally induced motion of molecular chains, create molecular entanglement and network structure, and help to improve melt strength [26], as observed in Figure 8. It is worth mentioning that the blends with high LPLA contents possess low free energy, difficult nucleation process, imperfect crystals, and dynamic equilibrium between molecular chain entanglement and unwinding due to respectable molecular chain fluidity, very few crystalline nucleation points, and extremely irregular molecular chain sequences. In the final analysis, the existing discrepancy in molecular weight leads to an alteration in the movement of the chain segment structure.

## 3. Materials and Methods

### 3.1. Materials

One PLA, grade 4032D, possessed a weight average molecular weight of 210 kg/mol and a melt index of 7 g/10 min (210 °C, 2.16 kg). Another PLA, grade 3251D, had a weight average molecular weight of 90 kg/mol and a melt index of 80 g/10 min (210 °C, 2.16 kg). Both PLAs were manufactured by NatureWorks LLC, Minneapolis, MN, USA, with densities and D-lactic acid contents of 1.24 g/cm^3^ and 1.4%, and melting temperatures of 155–170 °C. In the present investigation, high-molecular-weight 4032D was defined as HPLA, and low-molecular-weight 3251D was considered LPLA.

### 3.2. Sample Preparation

**Figure 9:**
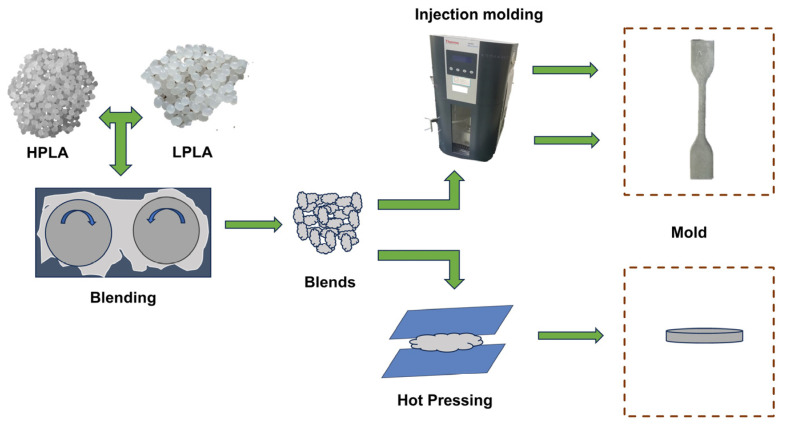
Schematic representation of the sample preparation process.

In order to remove the water in the raw materials, an appropriate amount of two kinds of PLA was weighed and dried in a blast drying oven at 50 °C for 8 h. The blend was obtained by melting and mixing at 170 °C and 50 r/min for 6 min by torque rheometer (PolyLab OS, HAAKE Co., Neustadt in Sachsen, Germany), and then various samples were obtained by injection molding or molding. For easy identification, all samples were labeled *x*HPLA/*y*LPLA, where x and y were the mass ratios of the HPLA and LPLA in the system, respectively, and were simplified to the *y*LPLA system. For the specific process, please refer to Figure 9.

### 3.3. Characterization

Differential scanning calorimetry (DSC; Q20, TA Instrument, New Castle, DE, USA): About 10 mg of the sample was weighed and a differential scanning calorimeter was utilized to heat the sample from 10 °C to 190 °C at a rate of 20 °C/min in the presence of N_2_ atmosphere; then, it was cooled for 3 min and heated the second time at 10 °C/min. X-ray diffraction analysis (XRD; LabX XRD-6000, Shimadzu, Kyoto, Japan): X-ray diffraction was employed, and monochromatic Cu-Kα radiation (λ = 0.15418 nm) was utilized as the X-ray source. To this end, the anode voltage and current were set to 40 kV and 30 mA, respectively, and the diffraction angle 2*θ* was scanned from 5° to 40° at the speed of 2°/min at room temperature. Polarized Microscopy (POM; Leica, Berlin, Germany) analysis: The sample film was prepared by hot press method and the crystal morphology of the sample was observed during isothermal crystallization with a polarized microscope on a platform controlled by a hot table. Scanning Electron Microscope (SEM; TM4000PlusⅡ, Hitachi, Tokyo, Japan): The surface morphology of the platinized fracture samples was imaged by a desktop scanning electron microscope with an acceleration voltage of 15 kV. Rheological Performance Test (DHR-2, TA Instrument, USA): The samples were pressed into small cylindrical discs by a flat vulcan press, and the rheological properties of the samples were appropriately measured by a rotary rheometer on a circular parallel plate (diameter 25 mm, gap 1.0 mm) at 180 °C under dynamic oscillation mode. The cutoff frequency was varied from 100 Hz to 0.01 Hz, and the strain was set at 1%. Thermogravimetric analysis (TGA; Q50, TA Instrument, USA): Approximately 3 mg of sample was weighed and heated from room temperature to 600 °C at 20 °C/min in the presence of a nitrogen atmosphere (flow rate 50 mL/min). Mechanical properties test (UTM4304, SUNS, Shenzhen, China): The tensile rate of 10 mm/min was tested by a universal testing machine at room temperature to carry out five tests and the average was calculated.

## 4. Conclusions

In this work, melt compounding was effectively utilized to create HPLA/LPLA blends with different molecular weights. The effects of low molecular weight on the quality of the mixture were methodically examined, and the following crucial results were achieved:

(1) DSC curves, POM images, rheological property curves, and SEM photographs all show that both the HPLA and LPLA have good compatibility.

(2) Due to the LPLA’s low molecular weight, strong fluidity, very slow crystallization rate, and the influence of amorphous components, the crystallization rate and spherulite growth size of the HPLA during the isothermal crystallization process reduced with growing the LPLA content, as did the crystallinity. The decrease in the peak intensity of XRD reveals that LPLA essentially exhibits the role of diluent in the mixed system, which affects the crystallization process of HPLA. The total enthalpy of the blending system did not lessen with the increase in the LPLA in non-isothermal crystallization, revealing that the HPLA with a high melting point acts as a nucleating agent to promote the LPLA crystallization growth, resulting in the co-crystallization phenomenon of the system; the tensile strength of the 5LPLA blending system was reported to be the highest.

As a result, the main effects of the low-molecular-weight polylactic acid on the polylactic acid blend system are as follows: on the one hand, the pure LPLA amorphous component inhibits the spherulite growth of the HPLA crystalline component, and as a result, the crystallization capacity decreases. On the other hand, since the melting point of HPLA is higher than that of LPLA, HPLA exhibits the role of the nucleating agent in the system and promotes the crystallization behavior of the system. Additionally, the LPLA reduces the viscosity of the system, lowers the surface force as well as the XRD power peak, enhances the rheological properties of the system, and has a diluting effect on the melt of the blend system. The correlation between the crystallization of different molecular weights and kinetics and thermodynamics is also utilized in this paper to optimize the physical and mechanical properties of polylactic acid composites and opens new potential fully biodegradable applications such as food packaging, foaming, construction, and drug carriers.

## Figures and Tables

**Table 1:** The thermal performance parameters associated with the plotted results in Figure 1.

Sample	DSC					
	*T_g_* (°C)	*T_cc_* (°C)	*T_m_*_1_ (°C)	*T_m_*_2_ (°C)	Δ*H_cc_* (J·g^−1^)	Δ*H_m_* (J·g^−1^)
100HPLA/0LPLA	61.7	117.6	165.9	170.3	38.3	45.6
95HPLA/5LPLA	62.2	118.0	165.0	169.7	37.2	44.5
90HPLA/10LPLA	61.9	120.4	164.0	168.9	36.8	44.0
85HPLA/15LPLA	61.7	121.4	164.2	169.1	37.7	44.1
80HPLA/20LPLA	61.8	121.1	164.2	169.1	37.7	44.6
0HPLA/100LPLA	59.8	128.2	151.8	-	8.4	13.7

**Table 2:** Weight loss temperature of both pure PLA and HPLA/LPLA blend systems.

Sample	TGA				
	*T_onset_* (°C)	*T*_5%_ (°C)	*T*_50%_ (°C)	*T_max_* (°C)	Residue (%)
100HPLA/0LPLA	350.6	338.8	368.8	372.5	6.2
95HPLA/5LPLA	351.2	336.6	366.7	370.9	2.1
90HPLA/10LPLA	352.3	340.8	369.0	372.2	5.7
85HPLA/15LPLA	350.3	342.8	366.2	368.1	6.8
80HPLA/20LPLA	345.2	325.9	360.6	366.1	0.2
0HPLA/100LPLA	351.4	337.2	366.1	370.1	2.8

## Data Availability

Data are contained within the article.

## Abbreviations

PLA	Polylactic acid
DSC	Differential scanning calorimetry
TGA	Thermogravimetric analysis
DTG	Differential thermogravimetry
XRD	X-ray diffractometer
POM	Polarized optical microscopy
SEM	Scanning electron microscopy
Symbols	
wt%	Weight percent
*T_g_*	Glass transition temperature
*T_c_*	Crystallization temperature
*T_m_*	Melting temperature
*T_cc_*	Cold crystallization temperature
*X_c_*	Crystallinity
Δ*H_c_*	Change in enthalpy
*T_onset_*	Crystallization initiation temperature
*T_endset_*	Crystallization end temperature
*φ*	Cooling rate
*t*_1/2_	Crystallization half-life
*T*_1/2w_	Full width at half maxima
*T*_5%_	5% weight loss degradation temperature
*T*_50%_	50% weight loss degradation temperature
*T_max_*	Maximum degradation rate temperature
*t*	Time
*T*	Temperature
